# Short-term outcome of intracorporeal ileocolonic anastomosis in patients with visceral obesity

**DOI:** 10.1038/s41598-024-63966-0

**Published:** 2024-06-10

**Authors:** Fangliang Guo, Cong Xia, Zongheng Wang, Ruiqi Wang, Yue Meng, Qianshi Zhang, Shuangyi Ren

**Affiliations:** 1 Department of Gastrointestinal Surgery, The Second Affiliated Hospital of Dalian Medical University, Dalian, 116023 Liaoning People’s Republic of China; 2 China Medical University, Shenyang, 110122 Liaoning People’s Republic of China

**Keywords:** Intracorporeal ileocolic anastomosis, Laparoscopic right colectomy, Prolonged postoperative ileus, Visceral obesity, Colon cancer, Gastrointestinal cancer, Cancer, Gastrointestinal cancer

## Abstract

The primary objective of this study was to compare short-term outcomes between Intracorporeal ileocolic anastomosis (IIA) and extracorporeal ileocolic anastomosis (EIA) after laparoscopic right hemicolectomy in patients with visceral obesity. The secondary objective was to identify risk factors associated with prolonged postoperative ileus (PPOI) after laparoscopic right hemicolectomy. This single-center retrospective study analyzed visceral obesity patients who underwent laparoscopic right hemicolectomy for primary bowel cancer between January 2020 and June 2023. Patients were categorized into IIA and EIA groups based on the type of anastomosis, and a 1:1 propensity score-matched analysis was performed. A total of 129 patients were initially included in this study, with 45 patients in each group following propensity score matching. The IIA group had significantly longer anastomosis times (*p* < 0.001), shorter incision length (*p* < 0.001), and shorter length of stay (*p* = 0.003) than the EIA group. Meanwhile, the IIA group showed a shorter time to first flatus (*p* = 0.044) and quicker tolerance of a solid diet (*p* = 0.030). On multivariate analysis, postoperative use of opioid analgesics is an independent risk factor for PPOI (OR: 3.590 95% CI 1.033–12.477, *p* = 0.044), while IIA is an independent protective factor (OR: 0.195 95% CI 0.045–0.843, *p* = 0.029). IIA remains a safe and feasible option for visceral obesity patients. It is also associated with a quicker recovery of bowel function and shorter length of stay when compared to EIA. Additionally, IIA is an independent protective factor for PPOI.

## Introduction

Colorectal cancer ranks as the third most commonly diagnosed cancer in both men and women, with right-sided colon cancer often diagnosed at advanced stages^1^. As a standard surgical treatment, laparoscopic right hemicolectomy (LRC) has gained widespread adoption in the treatment of colorectal cancer due to the evidence of oncological safety and advantages in short-term surgical outcomes^2–4^. A consequent critical issue is that the restoration of intestinal continuity and functional integrity after bowel resection relies on safe and effective anastomotic techniques. Among these, intracorporeal ileocolic anastomosis (IIA) and extracorporeal ileocolic anastomosis (EIA) are commonly well-established techniques for LRC, with EIA being more prevalent albeit fraught with constant controversy.

Nowadays, IIA has garnered increasing attention with advancements in surgical instruments and techniques^5^. Some studies suggest that despite its technical challenges, IIA offers benefits such as faster postoperative recovery, reduced complications, and shorter hospital stays^5–7^. However, conflicting results have been reported in other studies^8–10^, leading to the ongoing debate regarding the superiority of IIA in short-term outcomes. Effective postoperative recovery, particularly the restoration of bowel function, is paramount in colorectal surgery. Normally, bowel function returns to baseline within 2–4 days^11^. However, delayed recovery, termed prolonged postoperative ileus (PPOI)^12^, can lead to discomfort, psychological distress, prolonged hospitalization, and increased costs^13,14^. Therefore, investigating factors influencing postoperative recovery, such as the choice of anastomotic technique, becomes imperative.

In addition, the increasing prevalence of obesity in the population means that physicians are more likely to encounter visceral obese (VO) patients^15^. It's important to note that VO is linked to increased operative technical risks, challenges, and complications when compared to non-obese patients^16,17^. Compared with normal patients, obese patients have heavier and shorter mesenteries. To date, there has been no study specifically addressing the use of IIA in VO patients. Thus, exploring the outcomes of IIA versus EIA after LRC in VO patients is essential for optimizing surgical approaches in this population.

Therefore, the primary objective of this study was to compare short-term outcomes between IIA and EIA after LRC in patients with VO. The secondary objective was to identify risk factors associated with PPOI after LRC.

## Material and methods

### Patient selection

We retrospectively obtained data from prospectively collected data recorded of patients who underwent LRC at the Second Affiliated Hospital of Dalian Medical University from January 2021 to June 2023. Inclusion criteria were as follows: (1) pathological diagnosis of adenocarcinoma; (2) VO (visceral fat area ≥ 100 cm^2^)^18^; (3) age ≥ 18 years. Exclusion criteria were as follows: (1) the presence of distant metastasis; (2) emergency surgery due to bleeding, perforation, or obstruction; (3) preoperative chemotherapy or chemoradiotherapy; (4) multiple tumors; (5) simultaneous surgery for other diseases. All procedures were performed by a surgeon (Shuangyi Ren) with experience performing more than 2000 laparoscopic surgeries.

### Surgical technique

The initial steps of both procedures were standardized. The surgical approach began with a medial-to-lateral movement of the right colon, followed by bowel resection and lymph node clearance. Notably, all patients underwent D3 lymph node dissection, ensuring comprehensive nodal clearance. Major vessels were meticulously managed using titanium clips for secure hemostasis.

In cases of EIA, the intestine was extracted by extending the incision of the camera port, which was located in the supraumbilical midline. Following bowel resection, an ileocolic side-to-side isoperistaltic anastomosis was performed using a linear stapler. Subsequently, a 3–0 V-Loc barbed knotless suture was employed to reinforce closing the defect with sutures of the anastomosis with a continuous double-layer suture before returning the bowel to the abdominal cavity.

**Figure 1:**
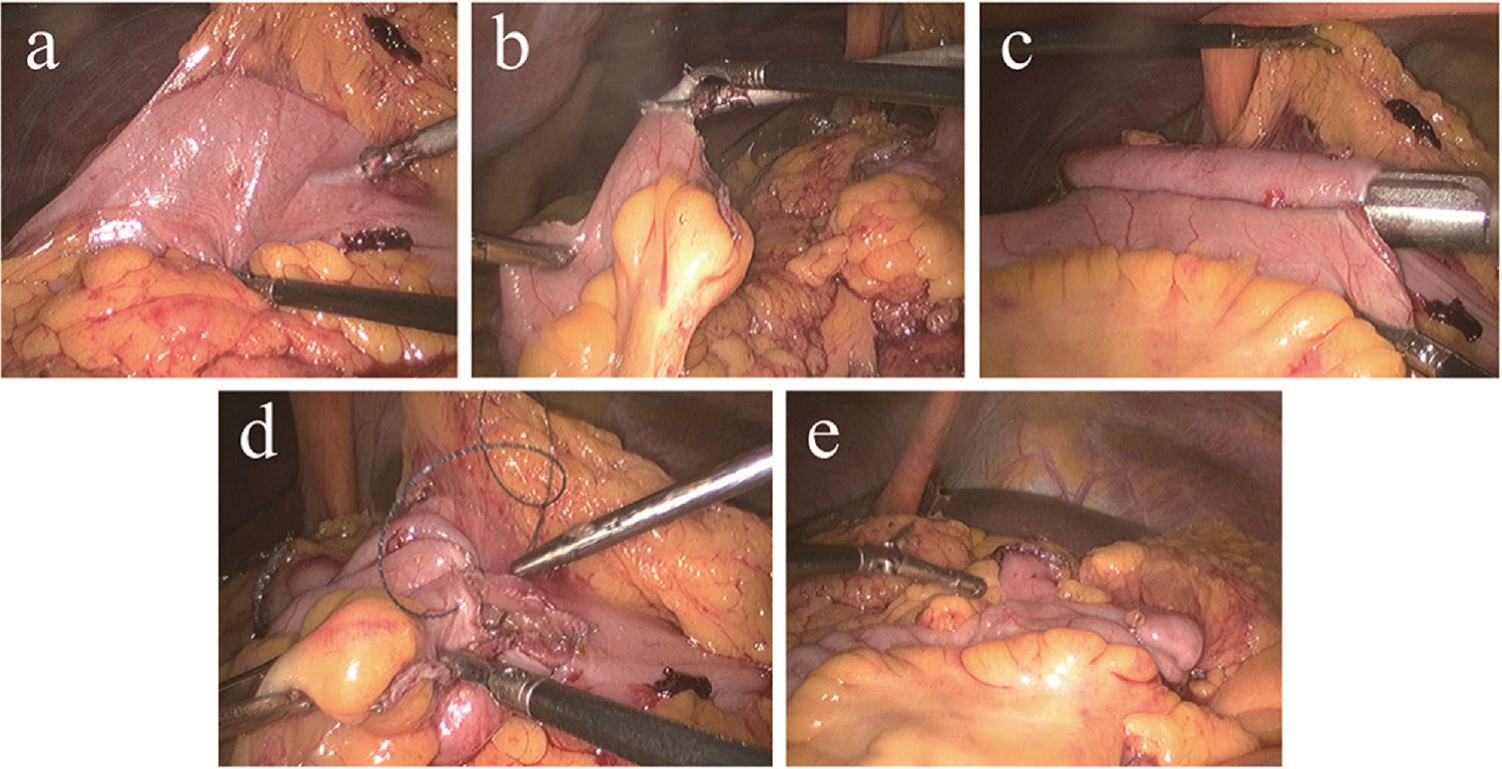
(**a**) One enterotomy was created near the transection side of the colon (**b**) One enterotomy was created on the terminal ileum (**c**) Inserted the jaws of the staples and stapled side-to-side ileocolonic anastomosis (**d**) Closed the common channel of the enterotomy with a barbed knotless suture by 3–0 V-Loc (**e**) Laparoscopic view of the completed anastomosis.

In cases of IIA, bowel resection and anastomosis were conducted intracorporeally. The ileocolic side-to-side isoperistaltic anastomosis was created using a linear stapler, with the closure of the enterotomy using laparoscopic sutures primarily. The specimen was removed by placing it in a specimen bag and extracted through a Pfannenstiel incision. The steps of IIA are summarized in Fig. 1.

### Perioperative management

Since 2017, our institution has been following the principles of Enhanced Recovery After Surgery (ERAS) and making adjustments according to clinical needs, as detailed in Table S1. All patients were treated by the same perioperative enhanced recovery care program. Preoperative evaluation included clinical examination, serological assessment, colonoscopy, and abdominal computed tomography (CT). The diagnosis of colon cancer was based on pathological results.

Pain was quantified using the visual analog scale (ranging from 0 to 10) to assess pain after surgery. The administration of postoperative opioid analgesics was also evaluated as a consequence of pain tolerance in patients. Complications were classified using the Clavien–Dindo classification.

### Variable and definition

**Figure 2:**
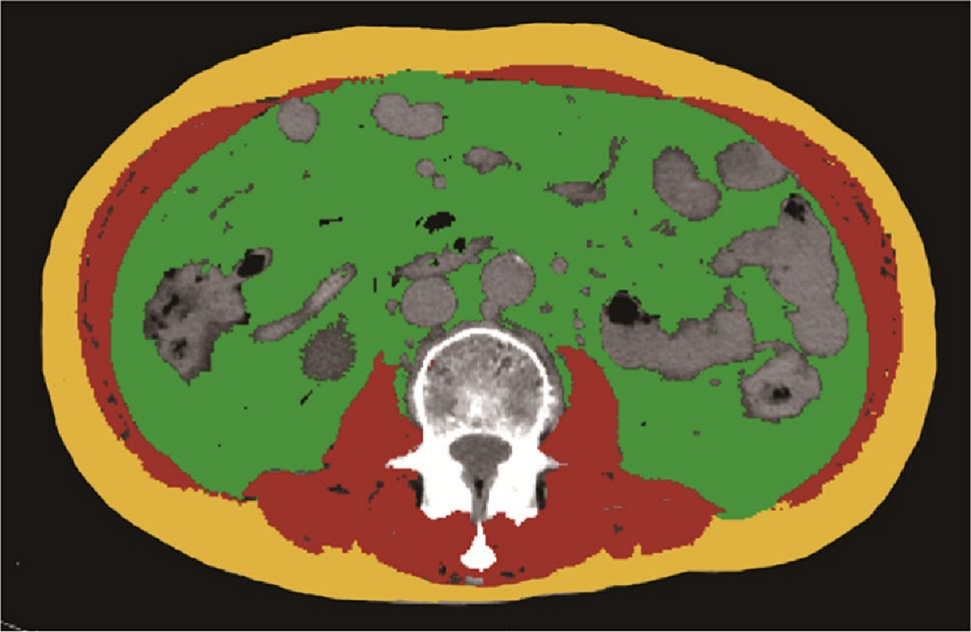
Body composition analysis carried out on CT images at the third lumbar vertebra level.

The CT images at the third lumbar vertebrae level were imported into slice Omatic 5.0 (Tomovision) medical imaging analysis software for body composition analysis (Fig. 2)^19^. Fat tissue was quantified by using recognized Hounsfield unit thresholds (− 190 to − 30 HU)^20,21^ and the visceral fat area was measured after differentiation from the subcutaneous fat area.

We adopted the definition of PPOI as proposed by Vather et al.^22^. PPOI was diagnosed when patients met at least two of the following five criteria on or after postoperative day 4: (1) nausea or vomiting; (2) inability to tolerate a solid oral diet over the last 24 h; (3) abdominal distension; d. absence of flatus over the last 24 h; (3) ileus noted on computed tomography (CT) scans.

### Statistical analysis

Statistical analysis was performed using Statistical Package for Social Sciences (SPSS) version 25 (IBM Corp, Armonk, New York, USA). The two groups were matched according to the anastomosis technique using a 1:1 nearest neighbor propensity score matching (PSM), with a caliper of 2 percent. Normally distributed continuous data were analyzed by Student's t-test and expressed as mean (± standard deviation [SD]); non-normally distributed data were analyzed by Wilcoxon rank-sum test and expressed as median (interquartile range [IQR]). Categorical data were compared using chi-squared test or Fisher’s exact test and expressed as n (%). Univariate logistic regression analysis was performed to initially assess associations of various indexes with PPOI. All indexes with *p* < 0.2 were included in the multivariate analysis. All of the statistical analyses were two-sided, and the statistical significance was set at *p* < 0.05.

### Ethics approval

This study was conducted in accordance with the ethical principles outlined in the 1964 Declaration of Helsinki. Ethical approval for this study was obtained from the Institutional Review Board Ethics Committee at The Second Affiliated Hospital of Dalian Medical University.

### Informed consent

Confirm that informed consent was obtained from all participants or their legal guardians.

## Results

### Patients' characteristics

**Figure 3:**
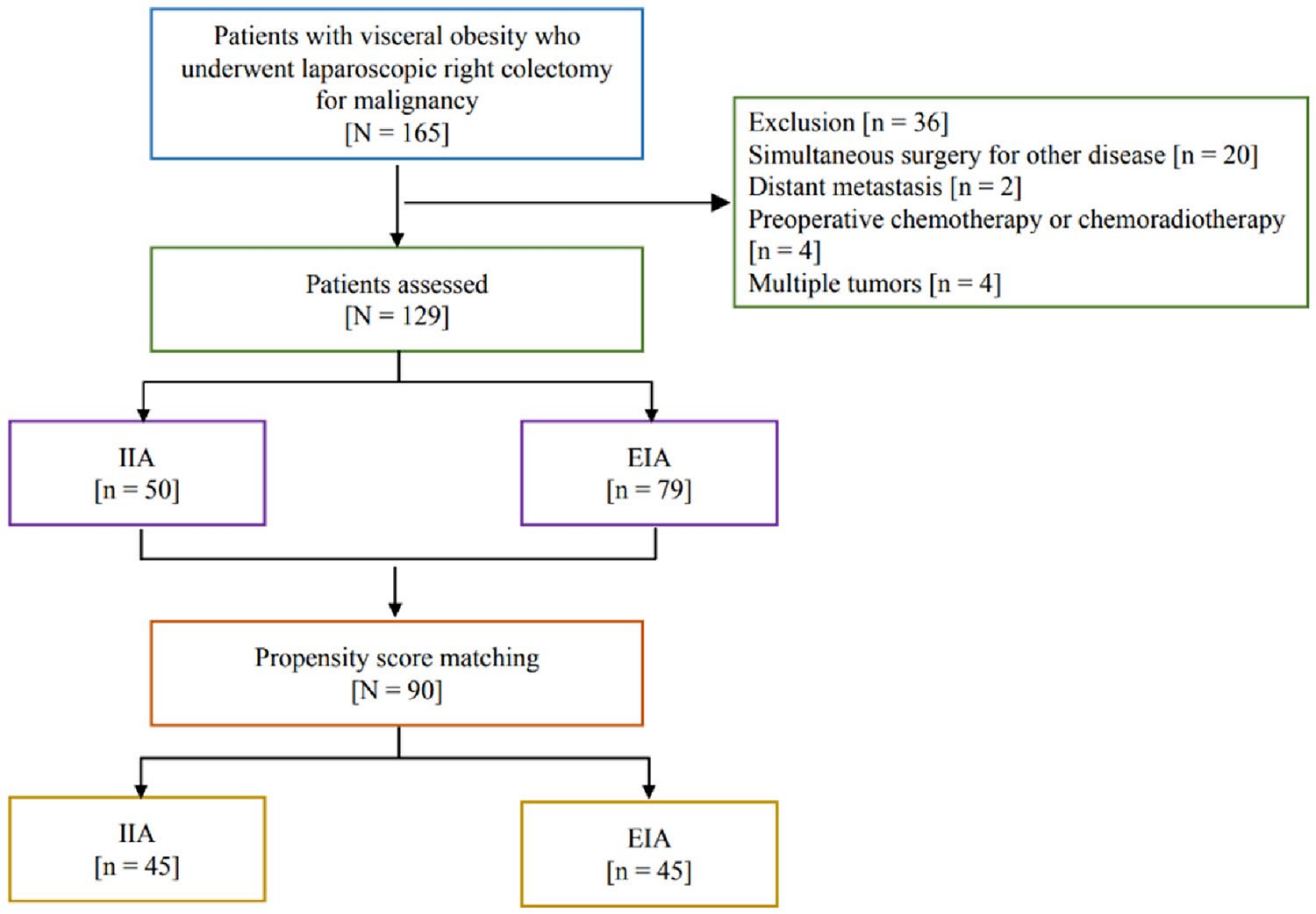
Flowchart of the patients assessed in this study.

A total of 165 patients were reviewed, of which 129 were included in our study (Fig. 3). In our cohort, 79 patients underwent EIA and 50 patients underwent IIA. Patients' characteristics according to the anastomosis technique are listed in Table 1. No significant differences were found between the EIA and IIA groups, except for Body mass index (BMI). After PSM, this study enrolled 45 patients in each group, and no significant differences were observed. The mean visceral fat area was 145.3 ± 27.3 cm^2^ in the IIA group and 148.2 ± 22.4 cm^2^ in the EIA group (*p* = 0.853).

**Table 1:** Demographic characteristics of patients before and after PSM.

	Before PSM			After PSM		
	IIA (n = 50)	EIA (n = 79)	*P*-value	IIA (n = 45)	EIA (n = 45)	*P*-value
Sex [n (%)]			0.476			0.673
Male	24 (48.0%)	43 (54.4%)		24 (53.3%)	22 (48.9%)	
Female	26 (52.0%)	36 (45.6%)		21 (46.7%)	23 (51.1%)	
Age [mean ± SD, years]	64.7 ± 11.8	66.7 ± 11.4	0.354	64.9 ± 11.6	65 ± 10.5	0.864
Smoking history [n (%)]	12 (24.0%)	25 (31.6%)	0.35	12 (26.7%)	15(33.3%)	0.490
Drinking history [n (%)]	6 (12.0%)	19 (24.1%)	0.092	6 (13.3%)	7 (15.6%)	0.764
Hypertension [n (%)]	13 (26.0%)	27 (34.2%)	0.328	11 (24.4%)	8 (17.8%)	0.438
Diabetes [n (%)]	9 (18.0%)	25 (31.6%)	0.087	8 (17.8%)	11 (24.4%)	0.438
Anaemia [n (%)]	15 (30.0%)	20 (25.3%)	0.56	14 (31.1%)	11 (24.4%)	0.480
Hypoproteinaemia [n (%)]	14 (28.0%)	26 (32.9%)	0.567	10 (22.2%)	10 (22.2%)	1.000
Previous abdominal surgery [n (%)]	11 (22.0%)	21 (26.6%)	0.557	11 (24.4%)	12 (26.7%)	0.809
ASA classification [n (%)]			0.624			0.393
I	26 (52.0%)	41 (51.9%)		23 (51.1%)	28 (62.2%)	
II	18 (36.0%)	24 (60.4%)		17 (37.8%)	11 (24.4%)	
III	6 (12.0%)	14 (17.7%)		5 (11.1%)	6 (13.3%)	
BMI [mean ± SD, kg/m^2^]	27.8 ± 3.3	26.5 ± 2.8	0.027	27.7 ± 3.2	26.9 ± 2.8	0.192
Visceral fat area [mean ± SD, cm^2^]	144.4 ± 26.5	149.9 ± 32.6	0.307	145.3 ± 27.3	148.2 ± 22.4	0.853
Tumour localization [n (%)]			0.329			0.930
Caecum	20 (40.0%)	23 (29.1%)		17 (37.8%)	15 (33.3%)	
Ascending colon	11 (22.0%)	13 (16.5%)		9 (20.0%)	10 (22.2%)	
Hepatic flexure	11 (22.0%)	27 (34.2%)		11 (24.4%)	10 (22.2%)	
Teansverse colon	8 (16.0%)	16 (20.3%)		8 (17.8%)	10 (22.2%)	

ASA: American Society of Anesthesiologists; SD: standard deviation.

### Operative and pathological parameters

Operative parameters are shown in Table 2. In comparison to the EIA group, the IIA group had a longer anastomosis time (*p* < 0.001), but the difference in overall operation time did not reach statistical significance (*p* = 0.071). Additionally, in the IIA group, all patients had their specimens extracted via Pfannenstiel incision, while in the EIA group, patients had their specimens extracted via the midline abdominal incision. The incision length was shorter in the IIA group (*p* < 0.001).

**Table 2:** Post-matching of operative and pathologic parameters of patients.

	IIA (n = 45)	EIA (n = 45)	*P*-value
Operation time [median (IQR), min]	155 (135–170)	148 (120–170)	0.071
Anastomosis time [median (IQR), min]	21 (18.5–23)	14 (13–16)	< 0.001
Intraoperative intravenous fluid [median (IQR), ml]	1300 (1200–1375)	1300 (1200–1350)	0.291
Estimated blood loss [median (IQR), ml]	65 (30–87.5)	60 (30–95)	0.849
Conversion [n (%)]	1 (2.2%)	6 (13.3%)	0.11*
Extraction site [n (%)]			
Midline	0	47 (100%)	< 0.001
Pfannenstiel	47 (100%)	0	
Incision length [median (IQR), cm]	5 (4–6)	6 (5–8)	< 0.001
Tumor diameter [median (IQR), cm]	5 (4–7)	5 (4–5.8)	0.39
Harvested lymph nodes [mean (IQR)]	24 (17–30.5)	21 (13.5–29.5)	0.258
Tumor stage [n (%)]			0.943
I	6 (13.3%)	5 (11.1%)	
II	21 (46.7%)	21 (46.7%)	
III	18 (40.0%)	19 (42.2%)	

IQR: Interquartile range *Using Fisher's exact test.

The analysis of pathological parameters demonstrated adequate results in both groups (Table 2). In detail, there is no significant difference between the two groups in terms of the length of tumor diameter (*p* = 0.390), number of harvested lymph nodes (*p* = 0.258), and tumor stage (*p* = 0.943).

### Short-term postoperative outcomes

Early postoperative outcomes are listed in Table 3. There were no statistically significant differences in the rates of overall complications (IIA vs. EIA: 11.1% vs. 20.0%, *p* = 0.344) and severe complications (IIA vs. EIA: 2.2% vs. 8.9%, *p* = 0.361) between the two groups. In addition, postoperative opioid analgesic requirements were also lower in the IIA group, but the difference did not reach significance (IIA vs. EIA: 26.7% vs. 44.4%, *p* = 0.078).

**Table 3:** Post-matching of postoperative outcomes of patients.

	IIA (n = 45)	EIA (n = 45)	*P*-value
Postoperative complications [n (%)]			
Any	5 (11.1%)	9 (20.0%)	0.245
Mild Complications (Clavien–Dindo < 3)	4 (8.9%)	5 (11.1%)	1*
Incision infection	1	1	
Anastomotic leakage	2	1	
Bleeding	1	0	
Intestinal obstruction	0	2	
Pneumonia	0	1	
Severe complications (Clavien–Dindo ≥ 3)	1 (2.2%)	4 (8.9%)	0.361*
Intraabdominal bleeding	0	1	
Anastomotic leakage	1	3	
30-day mortality [n (%)]	0	0	1
Vomit [n (%)]	7 (15.6%)	3 (6.7%)	0.108
Abdominal distension [n (%)]	16 (35.6%)	8 (17.8%)	0.057
Time to first flatus [median (IQR), days]	2 (2–2)	2 (2–4)	0.044
Time to first stool [median (IQR), days]	3 (3–3)	3 (3–4)	0.197
Time to tolerance of solids [median (IQR), days]	2 (2–3)	3 (2–4)	0.030
VAS at POD1 [n (%)]			0.058*
Mild (0–3)	33 (73.3%)	23 (51.1%)	
Moderate (4–6)	11 (24.4%)	17 (37.8%)	
Severe (7–10)	1 (2.2%)	5 (11.1%)	
Postoperative opioid analgesic [n (%)]	12 (26.7%)	20 (44.4%)	0.078
PPOI [n (%)]	5 (11.1%)	14 (31.1%)	0.037
Length of stay [median (IQR), days]	6 (5–7)	7 (6–9)	0.003

VAS: Visual analogue scale; POD: postoperative day *Using fisher's exact test.

There was no significant difference in the time to first stool between the two groups (*p* = 0.197). However, the IIA group showed a shorter time to the first flatus (*p* = 0.044) and quicker tolerance of a solid diet (*p* = 0.030). Additionally, PPOI occurred in 5 cases in the IIA group while 14 cases in the EIA group (*p* = 0.037). In terms of the length of stay, patients who underwent IIA observed a shorter length of stay (IIA vs. EIA: 6 [5–7] vs. 7 [6–9], *p* = 0.003).

### Univariate and multivariate logistic analysis of PPOI risk factors

Univariate and multivariate logistic analyses of factors influencing PPOI are reported in Table 4. Univariate analysis showed that age, smoking history, previous abdominal surgery, hypoalbuminemia, anastomosis technique, postoperative use of opioid analgesic, and estimated blood loss were potential predictors of PPOI. Subsequent multivariate logistic regression analysis showed that postoperative use of opioid analgesics is an independent risk factor for PPOI (OR: 3.590 95% CI 1.033–12.477, *p* = 0.044), while IIA is an independent protective factor (OR: 0.195 95% CI 0.045–0.843, *p* = 0.029).

**Table 4:** Univariate and multivariate analysis of factors associated with PPOI.

Variables	Categorization	Univariate analysis		Multivariate analysis	
		OR (95% CI)	*P*-value	OR (95% CI)	*P*-value
Age (years)	< 70	1		1	
	≥ 70	2.143(0.761–6.031)	0.194	3.762 (0.966–14.648)	0.056
Sex	Female	1			
	Male	0.707 (0.254–1.966)	0.506		
ASA classification	1	1			
	2	0.707 (0.221–2.262)	0.558		
	3	0.722 (0.137–3.811)	0.701		
Smoking history	No	1		1	
	Yes	1.990 (0.696–5.696)	0.199	1.291 (0.300–5.553)	0.732
Drinking history	No	1			
	Yes	1.837 (0.498–6.780)	0.361		
Previous abdominal surgery	No	1		1	
	Yes	2.178 (0.730–6.494)	0.163	1.810 (0.460–7.122)	0.396
Hypertension	No	1			
	Yes	0.645 (0.167–2.494)	0.525		
Diabetes	No	1			
	Yes	0.996 (0.288–3.445)	0.994		
Anaemia	No	1			
	Yes	1.627 (0.448–5.908)	0.46		
Hypoalbuminemia	No	1		1	
	Yes	2.603 (0.858–7.892)	0.091	3.169 (0.768–13.073)	0.111
Body mass index (kg/m2)	< 70	1			
	≥ 70	0.881 (0.277–2.805)	0.831		
Tumor stage	I	1			
	II	0.533 (0.113–2.526)	0.428		
	III	0.857 (0.187–3.937)	0.843		
Tumor location	Caecum	1			
	Ascending colon	1.548 (0.400–5.988)	0.527		
	Hepatic flexure	1.354 (0.354–5.173)	0.658		
	Teansverse colon	0.867 (0.189–3.981)	0.854		
Longest tumor diameter (cm)	< 5	1			
	≥ 5	0.724 (0.261–2.004)	0.533		
Anastomosis technique	EIA	1		1	
	IIA	0.277 (0.090–0.851)	0.025	0.195 (0.045–0.843)	0.029
Intraoperative intravenous fluid (ml)	< 1320	1		1	
	≥ 1320	2.463 (0.868–6.987)	0.090	1.944 (0.555–6.809)	0.289
Harvested lymph nodes	< 25	1		1	
	≥ 25	1.991 (0.714–5.557)	0.188	3.040 (0.671–13.779)	0.298
Estimated blood loss (ml)	< 100	1		1	
	≥ 100	2.517 (0.788–8.042)	0.119	3.186 (0.681–14.911)	0.141
Operation time (min)	< 180	1		1	
	≥ 180	1.948 (0.583–6.511)	0.279		
Conversion	No	1		2.529 (0.345–18.547)	0.362
	Yes	3.141 (0.638–15.450)	0.159		
Postoperative use of opioid analgesic	No	1		1	
	Yes	4.371 (1.505–12.693)	0.007	3.590 (1.033–12.477)	0.044

OR: Odds ratio; CI confidence interval.

## Discussion

Nowadays, with innovations in surgical instrumentation, IIA using linear staples and barbed suture material allows for the increasing application of total laparoscopic right hemicolectomies. However, due to the greater technical difficulty and associated risk of intra-abdominal infection with IIA, many surgeons still hesitate to use it for LRC, and EIA remains the technique of choice^23^.

Some researchers have suggested that IIA may offer potential advantages in obese patients due to its avoidance of the need for bowel exteriorization during anastomosis, which could be exceedingly challenging when dealing with a short and thick mesentery^24,25^. Vignali et al.^26^ conducted a comparison between IIA and EIA in obese patients (BMI > 30) and reported that IIA in obese patients is associated with a lower incidence of incisional hernias, and might reduce the risk of hospital readmission. In LRC, the procedure takes place within the visceral cavity and the surgical technique may be influenced by intra-abdominal anatomy and physical conditions. VO, defined as a large accumulation of visceral adipose tissue, is considered a more reliable indicator than BMI in reflecting obesity in Asians^27^. Specifically, excessive adipose tissue may hinder finding and maintaining an accurate anatomical plane during surgery and pose difficulties for surgical procedures, such as space limitation, constraints on visualization of mesenteric vascular structures, intraoperative stretching of the bowel, and lymph node dissection^28^. To our knowledge, the present study is the first to compare IIA and EIA in patients with VO.

The study results indicate that although the difference in surgical time between the two groups did not reach significance (*p* = 0.071), the IIA group required more time for anastomosis compared to the EIA group^8^. However, the increase in surgical time did not lead to a higher incidence of postoperative complications; the overall rate and severity of postoperative complications were similar in both groups. These findings align with the conclusions of several previous studies and confirm the non-inferiority of IIA^5,6^. It is noteworthy that in this study, all IIA procedures utilized a Pfannenstiel incision. Previous research has reported that Pfannenstiel incisions can reduce postoperative pain, minimize incisional infections, and decrease the occurrence of incisional hernias^28–30^. However, in our study, no association was observed between EIA and increased incisional infections, possibly due to the use of incision protectors during surgery.

In this study, the length of hospital stay was significantly shorter in the IIA group, indicating an overall better recovery compared to the EIA group. This superior recovery with IIA was characterized by less wound pain and improved bowel function^31–34^. The research findings indicate that PPOI is unavoidable even within the context of ERAS protocols^35^. Similarly, our study on PPOI remains informative for laparoscopic rectal surgery within the ERAS protocols. Notably, we observed that IIA was identified as an independent protective factor for PPOI. The following reasons may explain the low frequency of PPOI in patients who underwent IIA. First, for EIA, due to the anatomical characteristics of the transverse mesocolon, which is centrally located and relatively short, mobilization of the transverse colon is usually required to extract the colon. Experiments have shown that excessive intestinal processing can increase the inflammatory response of the intestinal muscular layer, which may lead to the occurrence of postoperative intestinal obstruction and slow down the recovery of intestinal function^36,37^. In contrast, IIA does not require exteriorization of bowel and mesentery, resulting in a lesser impact on gastrointestinal motility^20^.

Second, IIA is associated with less postoperative pain and less use of opioid analgesics^5^. In the present study, postoperative opioid analgesic was used in 44.4% of patients in the EIA group compared to 26.7% in the IIA group. The relationship between opioid use and PPOI has been well described by previous studies, and the present study arrived at a similar conclusion^38,39^. While opioid administration may vary depending on the type of anastomosis performed, the postoperative pain management strategies within the ERAS protocols are adaptable and diverse. Therefore, even with advancements in surgical techniques, the potential improvement in laparoscopic surgical outcomes by opioid-sparing approaches remains noteworthy^40^. Additionally, less pain allows the patient to mobilize and walk around the ward early, which may promote bowel movements.

Obviously, the present study has several limitations. The major limitation lies in its retrospective nature and lack of randomization, even if PSM was used to reduce the potential selection bias. Second, due to the retrospective nature of this study, data on total intravenous fluid administration were limited, which may have influenced our results. Despite the above-mentioned limitations, this study provides evidence for the advantages in postoperative recovery and safety of IIA in patients with VO.

## Conclusion

IIA remains a safe and feasible option for patients with VO. It is also associated with a quicker recovery of bowel function and shorter length of stay when compared to EIA. Additionally, IIA is an independent protective factor for PPOI.

### Supplementary Information

Supplementary Table S1.

## Data Availability

The data that support the findings of this study are available from the corresponding author upon reasonable request.
