# Supplementary material for: Short-term outcome of intracorporeal ileocolonic anastomosis in patients with visceral obesity
Source: Sci Rep. 2024 Jun 10;14:13247. doi: 10.1038/s41598-024-63966-0 (PMC11163010; doi:10.1038/s41598-024-63966-0)
Supplement: Supplementary file 1 — Supplementary Table S1. [file 41598_2024_63966_MOESM1_ESM.docx]

| Table S1 Items of the institutional ERAS protocol for colorectal surgery | |
| --- | --- |
| *Preoperative* |  |
| Preadmission education | The patient received preoperative counseling from a nurse and a physician, and a dedicated booklet including information on recovery goals and expectations about hospital stay |
| Carbohydrate drinks | Intake of a preoperative carbohydrate drink up until 2 h before anesthesia with at least 50 g carbohydrate in at least 400 mL fluid |
| No long-acting sedation | No preoperative long-acting sedative premedication |
| Thrombo-prophylaxis | Enoxaparin is used for antithrombotic prophylaxis postoperative days; Intermittent pneumatic compression |
| *Intraoperative* |  |
| Antibiotic prophylaxis | Cefminox 3.0g before incision |
| Epidural analgesia | Thoracic epidural analgesia started before surgical incision |
| Warming | Hypothermia prevention with active warming (air blanket) |
| Intraoperative fluid management | Intraoperative maintenance fluids excluding replacement of blood loss 3 ml/kg/h. If bowel preparation is used an extra 1000 ml of fluid is administered to cover losses. |
| Nasogastric tubes | No routine postoperative nasogastric tube |
| Abdominal drains | No resection-site drainage used |
| *Postoperative* |  |
| TED prophylaxis | TED prophylaxis with low molecular weight heparin |
| Postoperative analgesia | Epidural or PCA. Paracetamol, ibuprofen, and oxycodone-naloxone only for breakthrough pain |
| Early termination of IV fluid infusion | Termination of intravenous fluid infusion by the morning of POD 1 |
| Oral fluids | The patient received clear liquids on the day of surgery postoperatively |
| Oral nutritional supplements on POD 1 | The patient received one or more nutritional drinks on POD 1 |
| Mobilization at all on the day of surgery | Rising from bed 4 times more than 30 min |
| Bladder catheter | Removal on POD 1 |
| Stop epidural analgesia | Removal of thoracic epidural analgesia or PCA at POD 2 |

POD postoperative day, TED thromboembolic disease, PCA patient-controlled analgesia
